# Management of invasive candidiasis in the ICU: Challenges and advances (Review)

**DOI:** 10.3892/mi.2025.278

**Published:** 2025-10-24

**Authors:** Wael Ghaly Elmasry, Ahmed Mohamed Abdelbaky, Ahmed Hossameldin Ahmed Awad

**Affiliations:** 1 Department of Anesthesia, Mohammed Bin Rashid University of Medicine and Health Sciences and Intensive Care Unit, Rashid Hospital, Dubai, P.O. Box 4545, United Arab Emirates; 2 Department of Critical Care Medicine, Mohammed Bin Rashid University of Medicine and Health Sciences and Intensive Care Unit, Rashid Hospital, Dubai, P.O. Box 4545, United Arab Emirates

**Keywords:** invasive candidiasis, intensive care unit, antifungal resistance, diagnostic tools, novel antifungals, antifungal stewardship, *Candida* species, mortality, combination therapy, clinical guidelines

## Abstract

Invasive candidiasis (IC) is one of the principal causes of morbidity and mortality in critically ill patients, particularly in intensive care units (ICUs). Early diagnosis and prompt treatment are vital for achieving optimal outcomes for patients; however, factors such as delayed diagnosis, antifungal resistance and difficulty in managing critically ill patients make this difficult. The present review describes some of the major challenges involved in the management of IC in ICU settings, including delays in diagnosis, outbreaks of multi-drug-resistant *Candida* species and high mortality rates. Improvements in innovative diagnostic methods, including rapid molecular testing, as well as developments in antifungal therapy, such as rezafungin and ibrexafungerp, help improve patient outcomes. In addition, antifungal stewardship, prevention and combination therapy enhance the management of this complex infection. The present review also discusses clinical guidelines emphasizing the need for early empirical therapy, species-specific definitive treatment, and proper treatment duration. Continuous research toward new diagnostic procedures, antifungal agents and vaccines is essential to overcoming the challenges posed by IC in critically ill patients.

## 1. Introduction

Invasive candidiasis (IC) is a life-threatening fungal infection that is caused mainly by *Candida albicans* and other non-albicans *Candida* (NAC) species, including *Candida glabrata*, *Candida parapsilosis*, *Candida tropicalis* and *Candida krusei*. It manifests as candidemia or disseminated infection involving multiple organs, such as the liver, spleen, heart and central nervous system. Diagnosis is complicated due to non-specific symptoms such as fever, hypotension and multi-organ dysfunction. Blood cultures are the gold standard for diagnosis, but have limited sensitivity. The usual treatment involves the use of echinocandins or fluconazole, while the surgical removal of infected medical devices is also pivotal for patient management purposes (1,2).

IC is associated with a very high mortality in intensive care units (ICUs) of 30-50%, which necessitates rapid identification and treatment with appropriate antifungals. The emergence of antifungal resistance among NAC species has complicated management. Thus, infection control strategies, antifungal stewardship programs, catheter care and the timely removal of infected central venous lines are required to reduce the incidence of IC in ICUs. Among the common fungal infections acquired in ICUs worldwide, *Candida* species rank 4th among bloodstream pathogens encountered by hospitalized patients, with many being NAC species resistant to fluconazole and other antifungals. The global incidence of IC exhibits a wide variation, with higher rates in regions with limited healthcare resources and high antibiotic use. Another significant contributor to mortality is the delay in diagnosis and inappropriate antifungal therapy in patients in ICUs whose underlying conditions are already severe (1-4).

While the recent comprehensive review by Soriano *et al* (1) addressed current clinical challenges and unmet needs in invasive candidiasis across adult populations, the present review focuses specifically on critically ill patients in ICUs. The present review highlights ICU-specific risk factors, diagnostic limitations, therapeutic complexities due to organ dysfunction and invasive devices, and unique management strategies. By adopting this focused perspective, the present review complements prior research and provides intensivists with practical, ICU-centered insights into the management of invasive candidiasis (3).

## 2. Pathogenesis of invasive candidiasis

**Figure 1:**
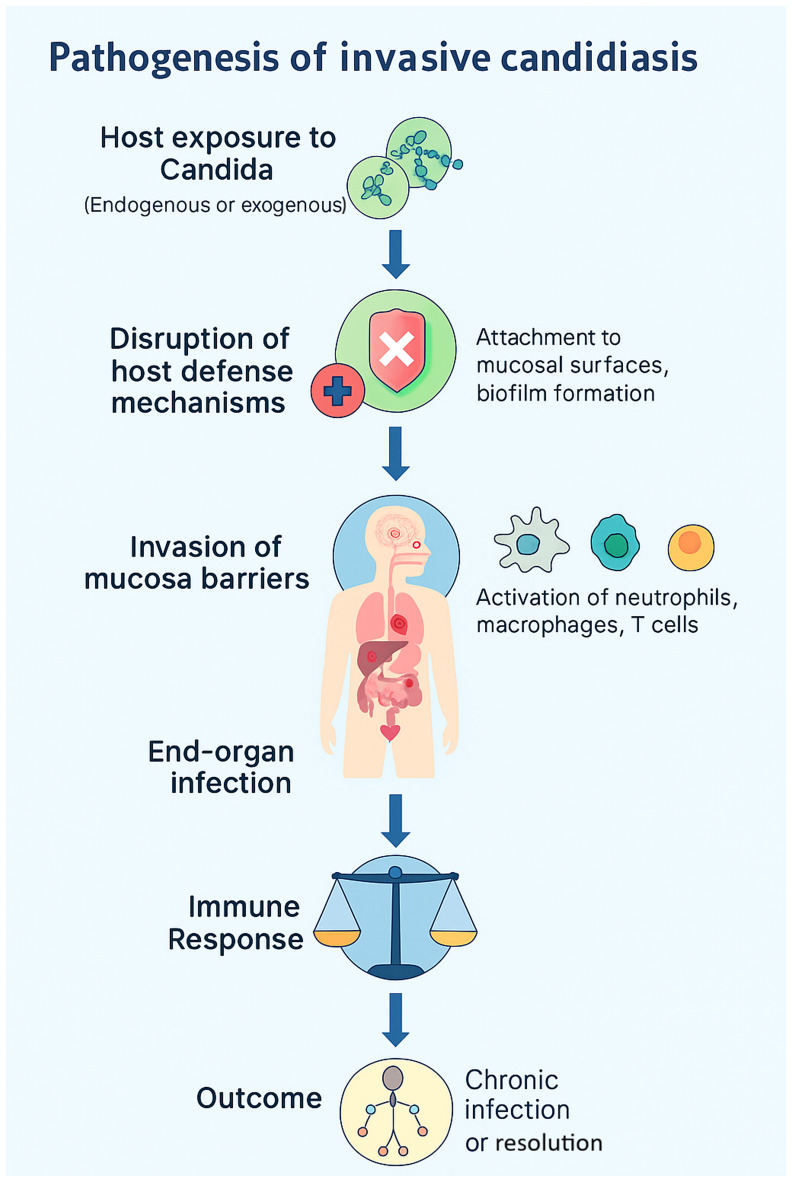
Pathogenesis of invasive candidiasis. Schematic flowchart illustrating the sequence from the initial colonization of mucosal surfaces and skin, through the disruption of host defenses (e.g., broad-spectrum antibiotics, immunosuppression, total parenteral nutrition), adhesion and biofilm formation on indwelling devices (central venous catheters), yeast-to-hyphae morphogenesis with the secretion of hydrolytic enzymes (secreted aspartyl proteases and phospholipases), mucosal invasion and translocation into the bloodstream (candidemia), hematogenous dissemination to distant organs (liver, spleen, kidneys and brain), immune evasion mechanisms (antigenic variation, oxidative stress resistance, modulation of host response), and the resulting clinical outcomes including sepsis, multi-organ failure, and high mortality rates in intensive care units.

IC, characterized by the ability to induce widespread systematic and immune evasion, is a disorder precipitated by the interaction of colonization, epithelial invasion, bloodstream dissemination and immune escape (5). *Candida* species are part of the normal commensal flora of the skin, gastrointestinal tract and other mucosal surfaces; these sites function as endogenous reservoirs rather than sources of external acquisition. Under normal conditions, host immunity and bacterial microbiota maintain a balanced state that prevents fungal overgrowth. However, factors such as immunodepression, broad-spectrum antibiotic use, critical illness and the insertion of indwelling medical devices disrupt this equilibrium, enabling *Candida* to shift from commensal to pathogen (4-6). The invasive process begins with opportunistic overgrowth in response to host defense alterations (as illustrated in Fig. 1), followed by adhesion to epithelial cells or medical devices through surface adhesins (5,6). A critical virulence step is the yeast-to-hyphal transition, which allows penetration of mucosal barriers. Hyphal forms secrete hydrolytic enzymes, including secreted aspartyl proteases and phospholipases, leading to tissue damage and deeper invasion (5). Once the mucosal barrier is compromised, *Candida* can translocate into the bloodstream, resulting in candidemia. Hematogenous dissemination permits colonization of distant organs such as the liver, spleen, kidneys and brain (4,5). Biofilm formation, particularly on central venous catheters, contributes to persistent infection by enhancing resistance to antifungal therapy and impairing immune clearance (4,6). *Candida* employs several immune evasion strategies, including antigenic variation, secretion of immunomodulatory molecules and resistance to oxidative stress (5,6). Host factors, such as neutropenia, corticosteroid therapy and impaired T-cell responses significantly increase the risk of systemic dissemination, culminating in sepsis, multiorgan failure and high mortality in critically ill patients (4-7).

### Risk factors

IC in patients in ICUs is strongly associated with multiple predisposing factors, including the prolonged use of broad-spectrum antibiotics, central venous catheterization, mechanical ventilation, parenteral nutrition, renal replacement therapy, corticosteroid therapy and underlying immunosuppression. Recent systematic reviews confirm that these risk factors significantly increase susceptibility to candidemia and invasive fungal disease in critically ill patients (6-8). A summary of the key ICU-specific risk factors for IC is presented in Table I.

### Symptoms

The clinical presentation of IC in patients in the ICU is usually non-specific, often mimicking bacterial sepsis. Common features include persistent fever despite broad-spectrum antibiotics, hypotension, signs of septic shock, multi-organ dysfunction, and occasionally, the focal involvement of organs such as the liver, spleen, or kidneys (4-9). These manifestations are summarized in Table II for clarity.

### Diagnosis

Diagnosis remains challenging due to the non-specific clinical features and the limited sensitivity of blood cultures. Culture-based methods remain the gold standard but require up to 72 h for results. Non-culture-based assays, including (1→3)-β-D-glucan (BDG), mannan antigen/anti-mannan antibody and PCR-based tests, provide a more rapid detection, while advanced platforms, such as the T2Candida panel allow for rapid species identification directly from blood samples (9-11). The strengths and limitations of available diagnostic modalities are summarized in Table III.

## 3. Management of invasive candidiasis

The management of IC in the ICU is built upon three essential principles: The timely initiation of empiric antifungal therapy, targeted treatment tailored to pathogen identification and susceptibility results, and effective source control. These pillars, supported by careful monitoring and supportive care, remain the cornerstone for improving the survival of critically ill patients. The early initiation of empiric therapy is crucial, as delays are consistently associated with increased morbidity and mortality rates. Echinocandins (caspofungin, micafungin and anidulafungin) are generally recommended as first-line agents in this setting, owing to their broad antifungal spectrum, fungicidal activity against the majority of *Candida* species and favorable safety profile. In hemodynamically stable patients without prior azole exposure and with a high likelihood of *Candida albicans* infection, fluconazole may be considered as an alternative. Amphotericin B, while highly effective, is generally reserved for patients with resistant species infection or intolerance to other agents, given its nephrotoxicity and infusion-related side-effects (12-14).

Once the infecting species and susceptibility profile are available, de-escalation to targeted therapy is essential. *Candida albicans* is usually susceptible to fluconazole, which remains an effective step-down option, whereas non-albicans species such as *Candida glabrata* and *Candida krusei* often necessitate continued echinocandin or amphotericin B therapy due to resistance patterns. This highlights the importance of routine susceptibility testing, which allows clinicians to optimize therapy and reduce the risk of treatment failure or resistance emergence (12-15).

The recommended duration of antifungal therapy is typically at least 14 days following the documented clearance of *Candida* from the bloodstream and the resolution of clinical signs and symptoms. However, deeper foci of infection, such as endocarditis, osteomyelitis, or intra-abdominal abscesses may require extended courses of therapy lasting several weeks. The removal of central venous catheters or other indwelling devices implicated as the source of infection is critical, as antifungal therapy alone is often insufficient in the presence of biofilm-associated colonization. Similarly, surgical drainage or debridement may be required for abscesses or localized invasive disease (12,15-17).

Supportive care plays an equally critical role in management. This includes hemodynamic stabilization, glycemic control (particularly in diabetic patients receiving corticosteroids), nutritional optimization and organ support measures, as needed. Regular monitoring with repeat blood cultures, imaging analyses to evaluate persistent infection, and the laboratory surveillance of renal and hepatic function is essential to assess the treatment response and mitigate drug-related toxicities (14-17).

In summary, the optimal management of invasive candidiasis in the ICU requires an integrated approach that combines the rapid initiation of empiric antifungal therapy, adjustment based on pathogen-directed susceptibility, effective source control and comprehensive supportive care. Despite these well-established principles, clinicians continue to face substantial barriers that compromise timely diagnosis, appropriate therapy and patient outcomes.

## 4. Challenges in the management of invasive candidiasis in the ICU

The management of IC in critically ill patients remains particularly challenging due to the interplay between host factors, diagnostic limitations, therapeutic complexity and healthcare system constraints. Patients in ICUs frequently present with multiple organ dysfunction (renal, hepatic, or cardiovascular), that not only complicates the course of illness, but also alters antifungal pharmacokinetics, necessitating dose adjustments and vigilant monitoring for toxicity. Immunocompromised states, whether due to hematologic malignancies, organ transplantation, corticosteroid therapy, or HIV infection, further diminish host defenses and predispose patients to persistent or recurrent infections. Delayed diagnosis continues to be one of the most crucial obstacles. Clinical manifestations are non-specific fever, hypotension, or an unexplained clinical deterioration and are easily confounded with bacterial sepsis or other ICU-related infections. While blood culture remains the gold standard for candidemia, it is a lengthy process, requiring 48-72 h for results. This diagnostic delay, compounded by the limited availability of advanced molecular or antigen-based tests in numerous ICUs, often postpones the initiation of effective antifungal therapy, thereby worsening outcomes (17,18).

Antifungal resistance represents another growing concern, particularly among NAC species, such as *Candida glabrata* and *Candida krusei*, which exhibit a reduced susceptibility to azoles. Although echinocandins have emerged as effective alternatives, resistance, albeit uncommon, has been reported. The absence of oral echinocandin formulations also complicates step-down therapy and prolongs outpatient management. Drug-drug interactions and polypharmacy further complicate the care of patients in ICUs, as antifungal agents may potentiate toxicities or alter the efficacy of other commonly administered drugs, including immunosuppressants, vasopressors and nephrotoxic antimicrobials (19-22).

The presence of invasive medical devices, including central venous catheters, endotracheal tubes and urinary catheters, also amplifies the risk of biofilm-associated infections that are poorly responsive to antifungal therapy alone. While device removal is critical for achieving a cure, this may be difficult or impossible in critically ill patients who depend on these interventions for survival. Such challenges, compounded by the lack of ICU-specific clinical guidelines for antifungal dosing, the duration of therapy and source control strategies, often leave clinicians to rely on individualized decision-making rather than standardized evidence-based protocols (22-24).

Beyond clinical complexities, IC imposes a significant economic burden, primarily due to prolonged periods of hospitalization in the ICU, repeated interventions and the high cost of antifungal agents (17,18,20). Prolonged periods of hospitalization, device-related procedures and the need for intensive supportive care substantially increase direct and indirect healthcare expenditures. Furthermore, the healthcare-associated nature of IC underscores the importance, but also the difficulty, of prevention in ICUs, where factors such as overcrowding, frequent use of invasive devices, and extended hospitalization create an environment conducive to nosocomial transmission (17,18,21).

The psychological and emotional toll on patients is another underrecognized dimension. Extended critical illness, invasive procedures, uncertain prognoses and the high risk of complications contribute to anxiety, depression and psychological distress in patients and their families, further compounding the burden of IC in ICU settings (20).

Collectively, these clinical, economic and psychosocial challenges highlight the urgent need for earlier diagnosis, individualized therapy, improved infection prevention, and ICU-specific clinical guidance to improve outcomes in this vulnerable population (17,18,21).

Notably, the growing recognition of these limitations has catalyzed significant innovations across diagnostics, therapeutics and preventive strategies. Novel diagnostic tools include rapid non-culture-based assays such as (1→3)-β-D-glucan, mannan antigen and anti-mannan antibody tests, PCR-based platforms, and the T2Candida panel. These modalities significantly reduce the time to detection compared with blood cultures, enabling earlier initiation of targeted therapy (19-24). The T2Candida panel, in particular, can detect *Candida* DNA directly from whole blood within 3-5 h with high sensitivity and specificity, representing a major advance in early diagnosis (23,24).

On the therapeutic front, newer antifungal agents and treatment strategies are reshaping management. Echinocandins remain first-line agents; however, the recent development of new triazoles, such as isavuconazole and investigational agents such as rezafungin and ibrexafungerp provides expanded options, including improved pharmacokinetics and oral formulations for step-down therapy (25-29). Combination antifungal therapy is being explored to overcome resistance among non-albicans *Candida* species (28,29). Therapeutic drug monitoring (TDM) for agents, such as voriconazole and posaconazole further enables individualized dosing in critically ill patients with altered pharmacokinetics (30-32).

In parallel, infection prevention and stewardship programs have become central components of management. These include strict catheter care protocols, antifungal prophylaxis in selected high-risk populations, and antifungal stewardship initiatives that promote judicious drug use and de-escalation based on local epidemiology and biomarker data (30,31,33-41). Collectively, these innovations in diagnostics, therapeutics and prevention represent a paradigm shift toward more rapid, tailored and multidisciplinary approaches to IC in the ICU (17-24,28-31).

## 5. Advances in the management of invasive candidiasis in the ICU

In recent years, notable advances have been made to address the challenges encountered in the management of IC, spanning from earlier diagnostic approaches to novel therapeutic strategies and multidisciplinary care models. Early recognition remains the cornerstone of improved outcomes, and several non-culture diagnostic tools have markedly shortened the diagnostic window compared with conventional blood cultures. Blood cultures, although considered the reference standard, have limited sensitivity (~50%) and require 24-72 h to yield positive results, with performance declining in deep-seated infections without candidemia (17,18). By contrast, non-culture assays enable earlier the initiation of antifungal therapy, often before overt clinical manifestations, thereby reducing diagnostic delays and potentially improving survival.

Among these, BDG testing demonstrates a pooled sensitivity of ~70-80% and a specificity of ~80-85%, with results available within hours; however, its specificity is reduced in the ICU due to false positives associated with hemodialysis, intravenous immunoglobulins, albumin and certain antibiotics, rendering it most useful as a rule-out test (19,20). Mannan antigen and anti-mannan antibody assays, when used in combination, provide improved sensitivity (~83%) and specificity (~86%), often yielding positive results several days before blood cultures, though their availability remains variable (21). PCR-based assays yield higher pooled sensitivity (~95%) and specificity (~92%) and provide rapid turnaround, but lack standardization and are constrained by costs and accessibility (22). Recently, the T2Candida panel, which uses magnetic resonance to directly detect *Candida* DNA from blood within 3-5 h, has demonstrated a sensitivity of ~89-100% and a specificity of ~96-99% for the most common species; however, it is limited by high costs, instrument availability and a restricted species panel (24). The practical utility of these assays lies in combining them with culture-based methods to accelerate rule-in or rule-out decisions, while ensuring species identification and susceptibility testing for definitive management.

Therapeutically, echinocandins have become the standard of care for empiric treatment due to their potent activity and favorable safety profile. The development of newer triazoles, such as isavuconazole, provides additional options, including oral formulations for step-down therapy. Combination regimens and novel antifungal agents under investigation are being explored to counter emerging resistance and broaden the therapeutic armamentarium (28,29). In parallel, pharmacokinetic and pharmacodynamic optimization has gained importance in critically ill patients, whose altered physiology often disrupts drug distribution and clearance. Therapeutic drug monitoring (TDM), particularly for agents, such as voriconazole, enables individualized dosing and enhances treatment precision, while minimizing toxicity (37-41).

Addressing biofilm-associated infections has also emerged as a key focus area. Biofilm formation on indwelling medical devices contributes to persistent infection and therapeutic failure; current research is investigating antifungal agents with biofilm-disruptive properties and innovative device technologies to reduce colonization and infection risk (4,6,8,12,13,28,29).

Complementary to pharmacologic advances, infection prevention and control measures play a pivotal role. These include antifungal stewardship programs, stricter catheter care protocols, and bundled prevention strategies, all of which have been shown to reduce ICU-acquired *Candida* infections (17,18,30,31). Stewardship initiatives emphasize judicious antifungal use, early de-escalation based on clinical and microbiological data, and the integration of local epidemiology and biomarkers into therapeutic decision-making, thereby reducing antifungal resistance and improving patient outcomes (8,28,30,31). Emerging therapies extend beyond antifungal drugs themselves. Immunomodulatory approaches, such as granulocyte colony-stimulating factor and experimental vaccines against *Candida* species are under investigation to augment host defenses, particularly in immunocompromised patients in the ICU (30,31). Furthermore, the adoption of multidisciplinary teams, including infectious disease specialists, intensivists, pharmacists and microbiologists has improved diagnostic accuracy, treatment optimization and stewardship.

Recent developments in predictive scoring systems, such as the Candida Score and APACHE II-based models, now allow clinicians to stratify patients and prioritize early therapy for those at greatest risk. Simultaneously, artificial intelligence and machine learning are being applied for predictive modeling, early detection and risk assessment of invasive candidiasis, providing a personalized and data-driven approach to ICU care (32,33).

Finally, host factors and comorbidities have become integral to management. Optimizing glycemic control, managing renal dysfunction, and minimizing unnecessary immunosuppression are essential strategies to reduce susceptibility and enhance response to therapy. Prophylactic antifungal use in select high-risk ICU populations, such as patients undergoing abdominal surgery, those with hematologic malignancies, or those undergoing transplantation remains an area of active investigation, although concerns regarding resistance, costs and toxicity necessitate cautious application. Collectively, these advances signal a paradigm shift toward earlier, more individualized and multidisciplinary care, integrating diagnostics, therapeutics, infection prevention and host optimization to improve the outcomes of patients with ICU (34-36).

## 6. Diagnostic modalities in the ICU

Blood culture remains the gold standard for the diagnosis of candidemia; however, its performance in critically ill patients is suboptimal. Sensitivity is estimated at only ~50%, and the time to positivity can range from 24-72 h, which delays targeted therapy and limits its value in deep-seated candidiasis without bloodstream involvement (17,18). Despite these drawbacks, cultures provide species identification and susceptibility testing, which remain indispensable for tailoring antifungal therapy.

To overcome the limitations of culture, several non-culture-based assays have been introduced. The BDG assay is among the most widely used biomarkers, with a pooled sensitivity of ~70-80% and specificity of ~80-85%, and a turnaround time of hours, once samples are processed. In the ICU, however, false positives are common due to exposure to hemodialysis membranes, intravenous immunoglobulin, albumin infusions and certain broad-spectrum antibiotics. As such, BDG is particularly valuable as a rule-out test in patients with low-to-intermediate pre-test probability rather than as a confirmatory tool (19,20).

Mannan antigen and anti-mannan antibody assays provide another non-culture diagnostic approach, with diagnostic yield improving significantly when both are combined. Multiple studies have reported a sensitivity of ~83-89% and a specificity of 86-90%, with serological positivity often preceding blood culture results by several days (21,42,43). However, test performance varies across *Candida* species, and availability remains inconsistent across ICU settings (21). Molecular methods, particularly PCR-based assays, have demonstrated a high pooled sensitivity (~95%) and specificity (~92%) for IC, with results available within 24 h. These assays allow for the much earlier initiation of antifungal therapy, although the lack of assay standardization, variable performance across platforms and higher costs limit widespread adoption (22).

A notable advancement in this field is the T2Candida panel, which detects Candida DNA directly from whole blood using magnetic resonance technology. The assay provides results within 3-5 h and covers the five most common *Candida* species, with a reported sensitivity of ~89-100% and specificity of ~96-99%. Despite these advantages, its clinical utility is tempered by high costs, limited species coverage and restricted availability in numerous ICUs (23). Taken together, culture- and non-culture-based diagnostics are best viewed as complementary rather than competitive. While blood cultures remain essential for definitive identification and susceptibility testing, non-culture assays, such as BDG, mannan antigen and anti-mannan antibody assays, PCR and T2Candida can greatly accelerate diagnosis, facilitate the earlier initiation or de-escalation of antifungal therapy, and improve the outcomes of critically ill patients. The optimal diagnostic approach in the ICU likely involves a multimodal strategy, integrating rapid biomarkers with traditional cultures and guided by clinical risk stratification to balance speed, accuracy and cost-effectiveness.

## 7. Dosing and therapeutic drug monitoring in the ICU

TDM has become an increasingly valuable tool in optimizing antifungal therapy in critically ill patients, in whom altered pharmacokinetics can result in either subtherapeutic exposure or toxicity (23,40). Although not all antifungals require TDM, it is particularly indicated for agents with narrow therapeutic windows, a high interpatient variability, or concentration-dependent toxicities (26,29). Voriconazole is the most well-established candidate, as trough concentrations are closely associated with both efficacy and toxicity. Subtherapeutic levels (<1-2 µg/ml) are associated with treatment failure, while supratherapeutic levels (>5-6 µg/ml) increase the risk of hepatotoxicity, neurotoxicity and visual disturbances; the generally recommended target trough range is 2-5 µg/ml (37,40,41). Posaconazole, particularly the oral suspension, demonstrates variable absorption in critically ill patients; TDM is therefore recommended, with prophylaxis targets of >0.7 µg/ml and treatment targets of >1.0-1.25 µg/ml (37,38,40). Itraconazole is another azole for which TDM is advised, with suggested trough levels >1 µg/ml to ensure efficacy (23,26). For 5-flucytosine, TDM is essential due to its narrow therapeutic index, and the risk of bone marrow suppression and hepatotoxicity at higher exposures. Target peak plasma concentrations are typically 30-80 µg/ml, with the avoidance of sustained levels >100 µg/ml (39,40). By candida.

In the ICU, where factors such as renal replacement therapy, extracorporeal membrane oxygenation and drug-drug interactions can alter antifungal disposition (23,40), TDM provides a practical means of tailoring therapy to achieve optimal exposure while minimizing toxicity. Its incorporation into routine practice for selected agents represents a critical advance in improving antifungal stewardship and clinical outcomes in critically ill patients with IC (8,28,40).

## 8. Prevention and infection control strategies

Prevention and control strategies for IC are particularly important for reducing incidence and improving patient outcomes in ICU settings. The routine screening and monitoring of at-risk patients, including those with central venous catheters, those on mechanical ventilation, or those treated with broad-spectrum antibiotics in recent days, may aid in identifying potential infections at an early stage. Accordingly, antifungal prophylaxis is frequently considered in patients who are considered high-risk, particularly when undergoing invasive surgery or whether they are in an immunocompromised state. Although antifungals for prophylaxis may be beneficial, the careful selection of patients needs to be undertaken so as not to expose any others unnecessarily and risk furthering resistance development. Strict infection control measures, apart from pharmacological interventions, need to be maintained. The standard precautions, such as hand washing, the sterilization of medical products, and the use of standard sterile techniques during invasive procedures, greatly limit the transmission of *Candida* species. The timely removal of central venous catheters when not medically necessary is another preventive strategy to reduce the risk of catheter-related bloodstream infection. Personalized antifungal stewardship programs in hospitals will limit antifungal use to situations in which it is truly required, while at the same time preventing the development of resistance and aiding in the implementation of proper empirical therapy on the basis of local resistance patterns. With these surveillance, prophylactical and infection prevention strategies, the incidence of developing IC in the ICU can be greatly reduced (37-40).

## 9. Conclusion and future perspectives

Future aspects and research gaps in the management of IC in ICU settings concern improving early diagnosis, optimizing treatment strategies and resolving antifungal resistance; one of the major areas of future studies could be the development of rapid, non-culture-based, advanced molecular and serological assays for the earlier detection of *Candida* infections, leading to timelier and more targeted treatment. Furthermore, personalized antifungal therapy could then be further improved, using genetic and pharmacokinetic profiling, as patients in the ICU may also have altered drug metabolism and clearance patterns. Research into novel classes of antifungal agents and into combination therapies could also pave the way, particularly in light of increasing resistance to azoles and echinocandins. Furthermore, the identification of biomarkers to predict resistance and outcomes in patients would enable more directed treatment strategies. Research on prevention strategies in high-risk ICU populations, particularly antifungal prophylaxis, de-escalation protocols and the management of indwelling devices, appears to be warranted. Furthermore, understanding host-pathogen interactions and the role of the microbiome in the development of IC may provide useful insights for prevention and treatment. Research into these issues would ultimately help to reduce the high morbidity and mortality rates associated with IC and benefit the management of patients in ICUs (36-37,40-42) (https://dig.pharmacy.uic.edu/faqs/2024-2/nov-2024-faqs/what-information-is-available-to-guide-the-practice-of-therapeutic-drug-monitoring-for-itraconazole-posaconazole-voriconazole-and-isavuconazonium-sulfate/).

In conclusion, the management of IC in patients in the ICU remains a complex challenge characterized by delayed diagnoses, difficulties in risk stratification, antifungal resistance and the requirement for pharmacokinetic adjustments. The persistently high mortality rates among patients in ICUs, despite advances in antifungal therapy and diagnostics, reflects the non-specific nature of clinical presentation and the critical baseline status of these patients. However, with the advent of rapid diagnostic techniques, novel antifungal agents and improved infection prevention strategies, there are promising avenues toward improving patient outcomes. Optimal results will be achieved through antifungal stewardship programs and multidisciplinary collaboration. Notably, unlike prior reviews that have addressed IC in broader adult populations (1-5,15,16), the present review provides an ICU-specific synthesis that integrates advances in diagnostics, antifungal pharmacology, therapeutic drug monitoring, infection control and predictive risk models. By tailoring insights to the critical care environment, the present review aimed to bridge the gap between general antifungal guidance and the practical realities faced in ICUs. Moving forward, research on personalized treatment protocols, novel antifungal therapies and prevention strategies will be of utmost importance to overcoming current challenges and improving the survival and quality of life of patients in ICUs.

## Figures and Tables

**Table I:** Risk factors: Key ICU-specific risk factors for invasive candidiasis, including device-related, pharmacological and host-related contributors.

Risk factor	Description
Immunocompromised state	Conditions including HIV/AIDS, cancer, and other disorders involving immunosuppressive therapies (e.g., corticosteroids, chemotherapy) reduce the ability of the body to oppose infections.
Diabetes mellitus	High blood sugar and insulin resistance may enhance *Candida* growth, particularly in patients with uncontrolled glucose levels.
Antibiotic use	Broad-spectrum antibiotics disrupt the normal microbiota, promoting *Candida* overgrowth.
Indwelling medical devices	Catheters, prosthetics, and central venous lines provide surfaces for *Candida* adherence, increasing the risk of infection.
Recent surgery or trauma	Surgical interventions or mucosal injuries may create entry points for *Candida* invasion into deeper tissues.
Prolonged periods of hospitalization	Longer hospitalization periods increase exposure to hospital-acquired infections and invasive devices.
Corticosteroid therapy	Long-term steroid use alters immune function, favoring *Candida* growth.
Pregnancy	Hormonal changes during pregnancy alter vaginal pH, predisposing women to vaginal candidiasis.
High estrogen levels	Estrogen (e.g., oral contraceptives) may displace normal vaginal flora, facilitating *Candida* overgrowth.
Obesity	Excess body weight, particularly abdominal fat, creates moist environments favorable for fungal growth.
Poor oral hygiene	Inadequate oral care predisposes to oral Candida overgrowth (oral thrush).
Malnutrition/poor diet	Micronutrient deficiencies impair immune function, increasing susceptibility to infection.
Age	Infants, elderly persons, and immunocompromised patients are more susceptible.
Chemotherapy/radiation	Cancer therapies weaken immune defenses, predisposing to infection.
Intravenous drug use (IVDU)	Contaminated needles or unsterile practices may introduce *Candida* into the bloodstream, causing systemic infection.

The information presented in this table has been taken from previous studies (1-4,6,18-21).

**Table II:** Symptoms: Common clinical features of invasive candidiasis in critically ill patients, highlighting non-specific symptoms that often mimic bacterial sepsis.

Symptom type/site	Symptoms
Systemic symptoms	Persistent fever, chills, hypotension, tachycardia, fatigue, sepsis-like symptoms (severe cases).
Bloodstream Infection (candidemia)	Persistent fever, positive blood cultures for *Candida*, sepsis (fever, chills, low blood pressure).
Endocarditis (heart)	Fever, heart murmurs, embolic phenomena (e.g., stroke, skin lesions).
Ophthalmic (endophthalmitis)	Vision changes, eye pain, redness.
Renal (kidneys)	Fever, flank pain, dysuria, hematuria.
Hepatosplenic candidiasis	Abdominal pain, jaundice, hepatomegaly (enlarged liver).
Peritonitis (abdomen)	Abdominal tenderness, distension, fever.
Pulmonary (lungs)	Respiratory distress if lungs are involved.
Gastrointestinal symptoms	Nausea, vomiting (if digestive tract is affected).

The information presented in this table has been taken from previous studies (1,2,4,6-7,21).

**Table III:** Diagnostic modalities for invasive candidiasis: Performance characteristics, turnaround time and limitations in intensive care unit settings.

Diagnostic method	Description
Clinical Evaluation	Detailed history and physical examination, particularly in immunocompromised patients (cancer, diabetes, transplant).
Blood cultures	Gold standard for candidemia; samples collected before antifungal initiation.
Urine culture	Used in suspected renal involvement (candiduria).
Tissue biopsy and culture	Obtained from affected organs (liver, kidney, heart) for definitive diagnosis.
PCR testing	Detects *Candida* DNA in blood or body fluids.
Serological tests	Mannan/anti-mannan antibodies and β-D-glucan test (fungal cell wall component).
Imaging analyses	Computed tomography scan or chest X-ray for pulmonary or abdominal involvement.
Endoscopy	Used for gastrointestinal candidiasis to visualize mucosal lesions.
Microscopic examination	Direct microscopy of tissue/fluid (e.g., abscess or peritoneal fluid) for yeasts/pseudohyphe.
Antigen detection	Detection of *Candida* antigens in serum or body fluids, adjunctive diagnostic tool.

The information presented in this table has been taken from previous studies (4,6-7,21-23,37-41).

## Data Availability

Not applicable.
